# Joint association of genetic risk and accelerometer-measured physical activity with incident coronary artery disease in the UK biobank cohort

**DOI:** 10.1371/journal.pone.0304653

**Published:** 2024-06-13

**Authors:** Robert C. Schell, William H. Dow, Lia C. H. Fernald, Patrick T. Bradshaw, David H. Rehkopf

**Affiliations:** 1 Analysis Group, Inc., Menlo Park, CA, United States of America; 2 Division of Health Policy and Management, School of Public Health, University of California, Berkeley, CA, United States of America; 3 Department of Demography, University of California, Berkeley, CA United States of America; 4 Division of Community Health Sciences, School of Public Health, University of California, Berkeley, CA, United States of America; 5 Division of Epidemiology & Biostatistics, School of Public Health, University of California, Berkeley, Berkeley, CA, United States of America; 6 Department of Epidemiology and Population Health, Stanford University, Palo Alto, CA, United States of America; University of Milan, ITALY

## Abstract

Previous research demonstrates the joint association of self-reported physical activity and genotype with coronary artery disease. However, an existing research gap is whether accelerometer-measured overall physical activity or physical activity intensity can offset genetic predisposition to coronary artery disease. This study explores the independent and joint associations of accelerometer-measured physical activity and genetic predisposition with incident coronary artery disease. Incident coronary artery disease based on hospital inpatient records and death register data serves as the outcome of this study. Polygenic risk score and overall physical activity, measured as Euclidean Norm Minus One, and intensity, measured as minutes per day of moderate-to-vigorous intensity physical activity (MVPA), are examined both linearly and by decile. The UK Biobank population-based cohort recruited over 500,000 individuals aged 40 to 69 between 2006 and 2010, with 103,712 volunteers participating in a weeklong wrist-worn accelerometer study from 2013 to 2015. Individuals of White British ancestry (n = 65,079) meeting the genotyping and accelerometer-based inclusion criteria and with no missing covariates were included in the analytic sample. In the sample of 65,079 individuals, the mean (SD) age was 62.51 (7.76) and 61% were female. During a median follow-up of 6.8 years, 1,382 cases of coronary artery disease developed. At the same genetic risk, physical activity intensity had a hazard ratio (HR) of 0.41 (95% CI: 0.29–0.60) at the 90^th^ compared to 10^th^ percentile, equivalent to 31.68 and 120.96 minutes of moderate-to-vigorous physical activity per day, respectively, versus an HR of 0.61 (95% CI: 0.52–0.72) for overall physical activity. The combination of high genetic risk and low physical activity intensity showed the greatest risk, with an individual at the 10^th^ percentile of genetic risk and 90^th^ percentile of intensity facing an HR of 0.14 (95% CI: 0.09–0.21) compared to an individual at the 90^th^ percentile of genetic risk and 10^th^ percentile of intensity. Physical activity, especially physical activity intensity, is associated with an attenuation of some of the risk of coronary artery disease but this pattern does not vary by genetic risk. This accelerometer-based study provides the clearest evidence to date regarding the joint influence of genetics, overall physical activity, and physical activity intensity on coronary artery disease.

## Introduction

Coronary artery disease (CAD) is a leading cause of death and disability worldwide [1, 2]. Both physical activity and genetic risk play a crucial role in its development [3, 4]. Decades of evidence demonstrate the importance of overall physical activity, referring to total physical activity accumulated, and intensity, referring to the proportion of time spent at higher levels of exertion, in reducing the risk of CAD [4–7]. However, in recent years, large-scale studies with accelerometer-measured physical activity suggest both that the benefits of physical activity in reducing the risk of CAD may be greater than previously realized and overall physical activity and physical activity intensity may each contribute to this risk reduction [8–11].

While genetic susceptibility to CAD was established decades ago using twin studies, recent genome-wide association studies have identified millions of variants associated with CAD [3, 12, 13]. Methods of combining these variants have enabled the construction of polygenic risk scores that have improved researchers’ ability to understand the genetic risk of developing CAD [14, 15].

Several studies have explored the combined impact of genetic susceptibility and self-reported lifestyle factors, including physical activity, on cardiovascular diseases [9, 16–20]. Genetic risk and physical activity had independent associations with cardiovascular disease and jointly increased overall risk in each study. However, these studies relied on questionnaire-assessed physical activity defined either dichotomously or as quantiles.

This subjective measure of physical activity has several limitations. Questionnaire-assessed physical activity demonstrated a weaker correlation with physical activity than objective measures [21, 22]. This method also does not account for incidental physical activity throughout the day. Administering longer questionnaires to provide a more holistic view of an individual’s daily physical activity results in higher levels of misclassification [23, 24]. Even when administered by a trained professional, questionnaire-based techniques suffer from recall and social desirability bias and perform poorly for people of less advantaged sociodemographic backgrounds [25, 26]. These sources of bias may obscure the associations between physical activity, genetic risk, and incident CAD. Additionally, modeling physical activity dichotomously or in categories ignores the continuous relationship between physical activity and CAD risk [4, 10]. Because these categorical analyses group physical activity intensity and overall physical activity together, these previous efforts could not distinguish their relative importance [9]. One recent study explores the impact of replacing sedentary time with physical higher levels of physical activity and produces results broadly consistent with ours [27].

This study evaluated the extent to which objective overall physical activity and physical activity intensity, measured by a wrist-worn accelerometer and modeled continuously, can offset an individual’s genetic susceptibility to incident CAD in the UK Biobank [28]. We utilized the best performing polygenic risk score to date, allowing for more precise genetic risk stratification than in previous efforts. Secondly, we explored whether a gene-environment interaction exists between overall physical activity, physical activity intensity, and genetic risk.

## Methods

### Accelerometer cohort

We used the UK Biobank (application # 79654), a population-based cohort of over 500,000 individuals from England, Scotland, and Wales aged 40–69 at recruitment between 2006 and 2010. Follow-up time was censored at March 31^st^, 2016 in Wales, September 30^th^, 2021 in England, and July 31^st^, 2021 in Scotland. The data were accessed most recently on September 4, 2023, and the authors did not have access to personally identifiable information. This dataset contains information on genetics, health behaviors, socioeconomic status, and health status and is described in detail elsewhere [28]. Between 2013 and 2015, participants with an email address were invited except those in the North West region due to concerns about participant burden. Out of 236,519 invitees, a subsample of 103,712 individuals responded to an email recruiting them to wear a wrist-worn Axivity AX3 triaxial accelerometer continuously for seven days on their dominant wrist and provided data. We applied exclusion criteria used previously in this dataset and dropped participants who failed calibration through either insufficient or unreliable data, had implausibly high overall acceleration averages, had wear time under three days, or did not have 24 unique hours of wear in a 24-hour cycle [29, 30].

### Genotyping & imputation

Participants in the UK Biobank were genotyped using either the UK BiLEVE or the UK Biobank Axiom Array, which each genotyped over 800,000 single-nucleotide polymorphisms (SNPs). Using either the Haplotype Reference Consortium panel or the UK10k and 1000 Genomes phase 3 panels, additional SNPs were imputed [31]. Following standard genetic quality control criteria in this dataset, we dropped individuals who withdrew consent or were not genotyped, had a mismatch between genetic and reported biological sex, sexual aneuploidy, outliers for missingness or heterozygosity, and we limited the dataset to the maximal set of individuals not related by third degree or closer [32]. We also split the dataset by ancestry, with those of White British ancestry as the sample for the analyses. Other ancestry groups contribute too few cases for analysis.

### Polygenic score

We applied the most predictive polygenic risk score available for CAD [14]. This score was derived by obtaining weights from the largest European-ancestry focused GWAS excluding the UK Biobank; and used PRS-CS, a polygenic risk score prediction method utilizing a Bayesian framework and continuous shrinkage robust to varying genetic architecture. We screened out multi-allelic SNPs, restricted to SNPs with an INFO score greater than 0.6, and restricted minor allele frequency to at least 0.01, yielding 1,087,647 variants included in the score. We then applied the scoring file available on PGS Catalog to recreate the scores derived in the original study [33]. We transformed the score into zero mean and unit variance. The UK Biobank has approval from the North West Multi-Centre Research Ethics Committee (MREC) to obtain and disseminate data and samples from the participants (http://www.ukbiobank.ac.uk/ethics/), which covers the analyses in this study. Written informed consent was obtained from all of the participants.

### Physical activity measures

Previous researchers processed the raw accelerometer data in the UK Biobank by calibrating to local gravity, filtering out sensor noise and gravity, and detecting and imputing non-wear time data segments to calculate the Euclidean norm minus one (ENMO) [29, 34]. The average ENMO was summarized as an average proportion of daily time spent at different categories of intensity measured in milligravities (mgs) based on measurements taken every 5 seconds and serves as the measure of overall physical activity in this study. Previous studies have demonstrated that ENMO correlates strongly with actual physical activity conducted [10]. We define physical activity intensity as minutes per day spent conducting moderate-to-vigorous physical activity. This is measured as minutes spent with an ENMO greater than 125 mgs, a typical cutoff point for MVPA which corresponds to an activity level at or above a brisk walk [35]. Physical activity intensity can refer to the full spectrum of physical activity from sedentary time and light intensity physical activity to moderate to vigorous physical activity. We chose to focus on MVPA as the measure of physical activity intensity because of its interpretability for both researchers and the public and because of the widespread use of MVPA in public health messaging by organizations such as the American Heart Association [17]. A common criticism of using these definitions for overall physical activity and physical activity intensity is the potential for collinearity owing to the intensity variable being a subset of the overall measure. As a sensitivity analysis, we explore the correlation between these measures and repeat the main analyses using physical activity energy expenditure for physical activity volume and percent of physical activity energy expenditure at moderate-to-vigorous physical activity for intensity, which are less prone to collinearity, in **S1 Table** and **S1-S3 Figs in the S1 File**.

### Outcome definition

We defined CAD based on hospital inpatient episodes, surgeries, and deaths. Specifically, we used ICD-10 codes I20 to I25, I46, and R96 to determine CAD as a cause of death, ICD-10 codes I20.0, I21-I22, and ICD-9 codes 410 and 4110 to denote a CAD event in hospital inpatient records, and OPCS-4 codes K40 to K46, K49, K501, K75 and OPCS-3 code 3043 to denote a CAD-related surgery. We restricted to incident CAD by excluding individuals with an event prior to the start of accelerometer wear. **S4 Fig in the S1 File** shows the Kaplan-Meier plot for survival in the sample.

### Covariates

In several waves, participants self-reported information on diet, health behaviors, parental heart disease history, mobility, employment status, and educational attainment pertinent to this analysis. These questionnaires did not occur at the same time as accelerometer wear. To minimize the bias from this discrepancy, we chose the value of the covariates from the most recent wave of self-reported data before accelerometer wear began. Diet consists of several variables, including whether an individual often adds salt to their food, past day consumption of fruits and vegetables, and weekly consumption frequency of oily fish and processed meat. Educational attainment denotes whether a person has a university degree, any other degree, or no degree. Health behaviors include smoking status divided into never, previous, or current and alcohol consumption measured as frequency of consumption per week. Employment status is defined as whether an individual is currently employed, and mobility problems denotes whether an individual has indicated any issues walking. **S2 Table in the S1 File** shows how we created these variables from UK Biobank data fields. We controlled for the first 10 genetic principal components, region, biological sex, the Townsend index measuring material deprivation, and season of wear, which as static variables did not depend on the wave selected. We explored the impact of measured body mass index, average sleep duration measured as self-reported hours per night, and cholesterol and blood pressure medication, all potential mediators, as well as manual labor conducted for one’s occupation in the supplement.

### Statistical analyses

We fit a Cox proportional hazards model with age as the timescale to measure the association between overall physical activity, physical activity intensity, genetic risk, and incident CAD with time-to-event as the outcome of interest. The model stratified on biological sex, the only covariate violating the proportional hazards assumption based on Schoenfeld residuals. Because the functional form of overall physical activity and physical activity intensity’s relationship with CAD could be nonlinear, we assessed model fit between the exposures modeled linearly or as a restricted quadratic or cubic spline. The linear model performed best for both physical activity exposures according to BIC. We ran the model with ENMO and polygenic risk score as continuous exposures and an interaction term between these exposures controlling for sex and then the full covariate set. Hazard ratios and 95% confidence intervals were then calculated by decile of genetic risk and overall physical activity with the 90^th^ and 10^th^ percentile (highest risk), respectively, serving as the reference group. We restricted to the 10^th^ and 90^th^ percentiles of risk instead of the maximum and minimum to avoid interpreting results based on the sparsely populated extremes of the distributions. We ran a model with minutes per day of MVPA and polygenic risk score as continuous exposures with an interaction term and controlling for ENMO and adjusting for sex and then the full covariate set and repeated the decile-based analysis. In sensitivity analyses, we excluded cases occurring within the first year of accelerometer wear to minimize possible reverse causation and stratified by sex. We relied on complete case analysis but imputed via multivariate imputation by chained equations as a sensitivity analysis.

We explored whether genetic risk and overall physical activity and physical activity intensity interact to increase risk of incident CAD by fitting interaction terms between the PA exposures and the polygenic risk score. All analyses were performed using R 4.1.3 [36]. All code is available on GitHub at https://github.com/BobbySchell/Joint-Association-of-Genetic-Risk-with-Incident-CAD-and-Accelerometer-Measured-PA.

## Results

### Population characteristics

**Fig 1:**
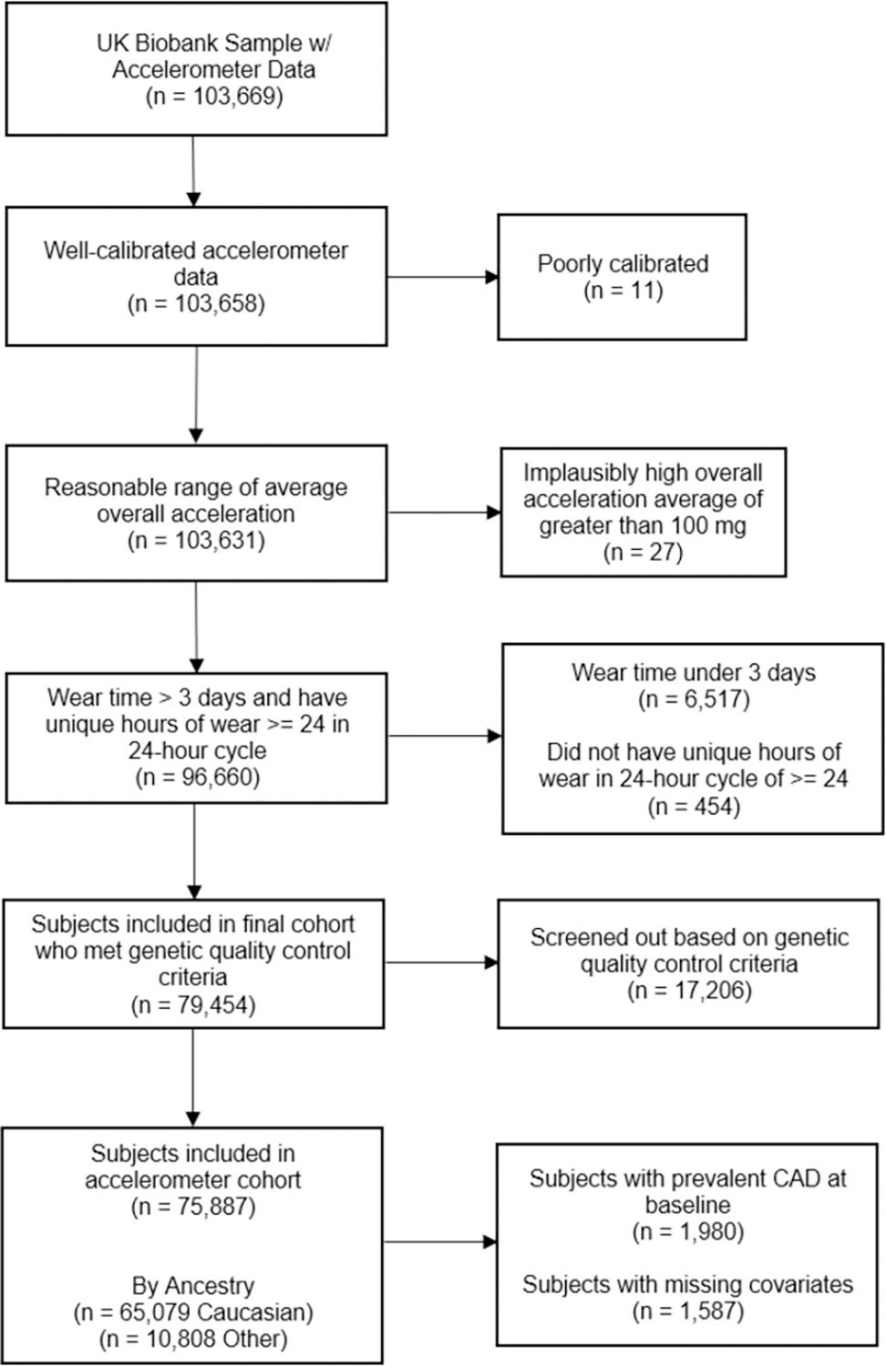
Subject exclusion criteria flowchart.

After screening individuals for valid accelerometer wear data, 96,660 participants remained in the study. We excluded 17,206 participants not meeting the genetic quality control criteria [32]. 1,587 participants had missing covariate data, and 1,980 had prevalent CAD at baseline, which left a final analytic sample of 75,887, among whom 65,079 participants were of White British ancestry as outlined in **Fig 1**. Compliance was high, with a median wear time of 6.9 days. **Table 1** shows the characteristics of the participants in our sample. The median follow-up time was 6.8 years with a total of 430,160 cumulative person-years and 1,368 CAD cases. The average age at baseline was 62.5 and participants in this sample were generally higher educated, less likely to smoke, and had lower levels of material deprivation than the larger population in the UK, which coheres with previous research [37]. Model 1 refers to the fully adjusted model and model 0 refers to the model adjusted for biological sex. **S3, S4 Tables in the S1 File** present the linear associations of genetic risk, physical activity, and incident CAD.

**Table 1:** Baseline characteristics.

Summary Statistics (n = 65,079; Incident CAD = 1368)		
*Variable*		
Follow-up Time, median (Interquartile range)		6.82 (6.29, 7.36)
Euclidean Norm Minus One (ENMO), mean (Standard deviation [SD])		28.03 (8.20)
Minutes per day of moderate-to-vigorous physical activity (mins/day MVPA), mean (SD)		73.30 (36.69)
Standardized Polygenic Risk Score, mean (SD)		0 (1.00)
Person-Years		430,160
Age in years, mean (SD)		62.51 (7.76)
**Highest Education Level, n (%)**		
	*University*	27,779 (42.69%)
	*Any Other Qualification*	32,076 (49.29%)
	*No qualification*	5,224 (8.03%)
Townsend Index, mean (SD)		-1.92 (0.08)
Currently Employed, n (%)		38,614 (59.33%)
Fruit & Vegetable Intake Quartile, mean (SD)		2.10 (0.59)
Weekly Alcohol Consumption Frequency, mean (SD)		3.02 (0.58)
Weekly Oily Fish Consumption Frequency, mean (SD)		1.10 (1.00)
Female, n(%)		36,790 (61.14%)
Parental History of Heart Disease, n (%)		26,737 (41.08%)
**Cigarette Smoking Status, n (%)**		
	*Never*	37,773 (58.04%)
	*Previous*	23,166 (35.60%)
	*Current*	4,140 (6.36%)
**Added Salt Intake, n (%)**		
	*Never*	39,573 (60.81%)
	*Rarely*	17,085 (26.25%)
	*Sometimes*	6,561 (10.08%)
	*Always*	1,860 (2.86%)
**Season Accelerometer Worn, n (%)**		
	*Fall*	19,329 (29.70%)
	*Spring*	14,810 (22.76%)
	*Summer*	17,086 (26.25%)
	*Winter*	13,854 (21.29%)
**Region, n(%)**		
	*England*	58,225 (89.47%)
	*Scotland*	4,322 (6.64%)
	*Wales*	2,532 (3.89%)
Mobility Limitations, n(%)		12,676 (19.48%)

### Overall physical activity & genetic risk percentile comparison

**Fig 2:**
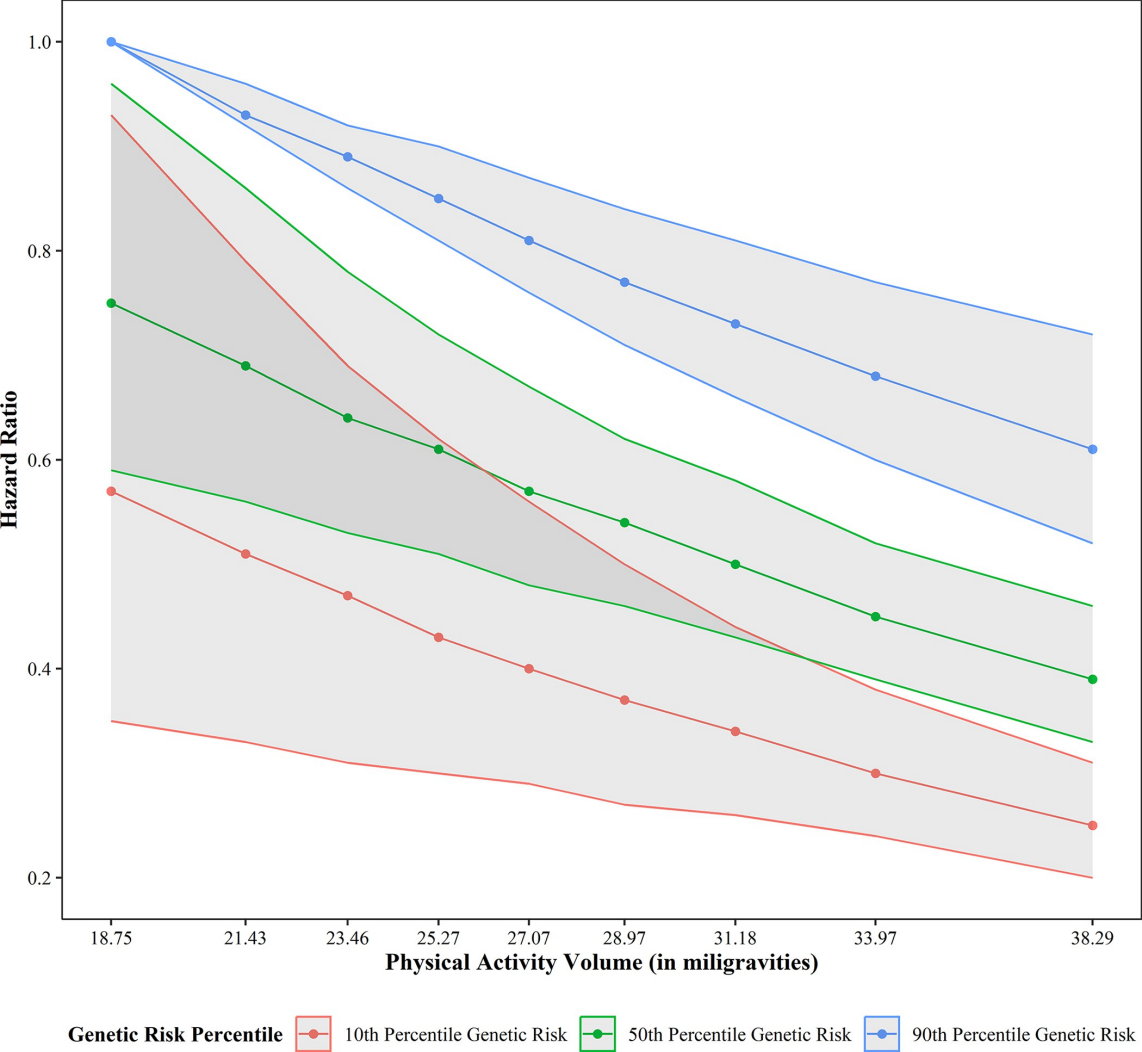
Overview of overall physical activity and genetic susceptibility results. Model 1 controlling for full set of covariates in main analyses. Results presented at 10^th^, 50^th^, and 90^th^ percentiles for the sake of clarity.

**Fig 2** plots the hazard ratios of participants at different genetic risk and ENMO percentiles, **Table 2** presents a subset of results, and **S5 Table the in S1 File** presents full results by decile. All results are for model 1 and within stratum hazard ratios refer to hazard ratio from a change in one variable at a set value of the other variable. Hazard decreases substantially at the highest levels of activity, with an individual at the 90^th^ percentile of ENMO (38.29 mgs) facing a 39% lower hazard (corresponding to a hazard ratio of 0.61) of incident CAD compared to an individual of the same genetic risk at the 10^th^ percentile of ENMO (18.75 mgs). Genetic risk has a stronger association as an individual at the 10^th^ percentile of genetic risk within the same ENMO stratum faces a 43% lower hazard of incident CAD than if they were in the 90^th^ percentile of genetic risk. While ENMO and genetic risk each have important independent associations with incident CAD, they combine to create the largest impact on risk of incident CAD. An individual at the 10^th^ percentile of genetic risk and 90^th^ percentile of ENMO faces a 75% lower hazard of incident CAD than the reference group.

**Table 2:** Overview of overall physical activity (as ENMO) and polygenic risk by percentile. Model 1 controlling for full set of covariates in main analyses. Results presented at 10^th^, 50^th^, and 90^th^ percentiles for the sake of clarity.

	ENMO		
Standardized Polygenic Score	10^th^ (18.75 mgs)	50^th^ (27.07 mgs)	90^th^ (38.29 mgs)
90^th^ (1.24 units)	REF	0.81 (0.76–0.87)	0.61 (0.52–0.72)
50^th^ (0.00 units)	0.75 (0.59–0.96)	0.57 (0.48–0.67)	0.39 (0.33–0.46)
10^th^ (-1.27 units)	0.57 (0.35–0.93)	0.40 (0.29–0.56)	0.25 (0.20–0.31)

### Physical activity intensity & genetic risk percentile comparison

**Fig 3:**
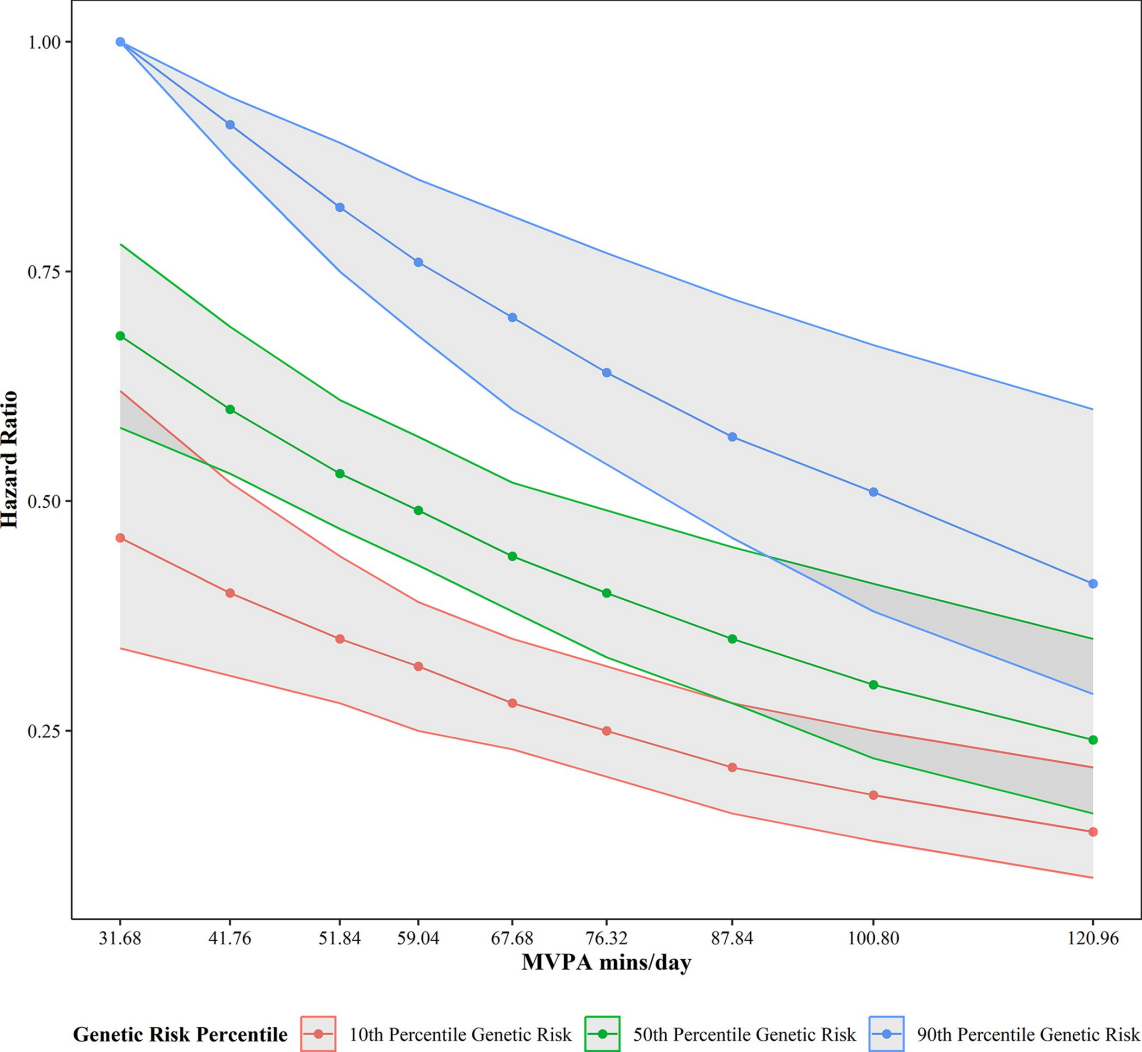
Overview of physical activity intensity and genetic susceptibility results.

Controlling for ENMO in model 1, **Fig 3**, **Table 3**, and **S6 Table in the S1 File** show that MVPA has a stronger association with incident CAD than ENMO. An individual at the 90^th^ percentile of MVPA (120.96 minutes per day) faces a 59% lower hazard (corresponding to a hazard ratio of 0.41) of incident CAD compared to an individual of the same genetic risk at the 10^th^ percentile (31.68 minutes per day). A participant at the 10^th^ percentile for genetic risk and 90^th^ percentile for MVPA faces an 86% lower hazard of incident CAD relative to an individual in the reference group. We explored possible interaction between overall physical activity and physical activity intensity and concluded that no significant interaction exists in this sample. We found no significant interactions between ENMO and genetic risk or MVPA and genetic risk, which is similar to what other studies found [9, 19].

**Table 3:** Overview of physical activity intensity (as minutes/day of MVPA) and polygenic risk by percentile.

	ENMO		
Standardized Polygenic Score	10^th^ (31.68 mins/day)	50^th^ (67.68 mins/day)	90^th^ (120.96 mins/day)
90^th^ (1.24 units)	REF	0.70 (0.60–0.81)	0.41 (0.29–0.60)
50^th^ (0.00 units)	0.68 (0.58–0.78)	0.44 (0.38–0.52)	0.24 (0.16–0.35)
10^th^ (-1.27 units)	0.46 (0.34–0.62)	0.28 (0.23–0.35)	0.14 (0.09–0.21)

### Sensitivity analyses

We repeated the main analyses using physical activity energy expenditure for physical activity volume and percent of physical activity energy expenditure at moderate-to-vigorous physical activity for intensity in **S1 Table** and **S1-S3 Figs in the S1 File** to determine how strongly collinearity affected the results. We excluded individuals with cases occurring within the first year of follow-up in **S5, S6 Figs in the S1 File**, reran the analyses with multivariate imputation by chained equations in **S7, S8 Figs in the S1 File**, and added potential mediators and occupation into the model with results in **S9-S12 Figs in the S1 File**. None of these choices substantially affected the results. We also stratified the models by sex in **S13, 14 Figs in the S1 File.** While the results for males closely matched the main results, the polygenic score’s effect was statistically insignificant for females.

## Discussion

### Overview of principal findings

In this study of 65,079 participants from the UK Biobank, genetic risk was associated with a higher risk of incident CAD regardless of overall physical activity or physical activity intensity. Overall physical activity and physical activity intensity each had significant independent associations with incident CAD, with physical activity intensity demonstrating the strongest association. While low overall physical activity and physical activity intensity were associated with an increased risk of CAD within a genetic risk stratum, low levels of overall physical activity and physical activity intensity combined with high genetic risk were associated with the greatest risk of incident CAD. This suggests that physical activity behavior may attenuate some of the high genetic risk of CAD. Specifically, an individual at the 90th percentile of genetic risk and overall physical activity or physical activity intensity faced a 39% or 59% lower hazard of CAD compared to if they also had 10th percentile levels of overall physical activity or physical activity intensity, respectively.

### Comparison with existing literature

Because previous studies discretize subjective physical activity, a direct comparison to estimates from the existing literature is not possible. However, the estimates for physical activity’s association with cardiovascular diseases in Said, *et al*. and Tikkanen *et al*. appear consistent with this study in size and direction of association [9, 19]. Zaccardi *et al*., rely on self-reported walking pace as the measure of physical activity and show that this has a large association with CAD, which is also consistent with our stronger results for physical activity intensity [20]. Because none of the above studies separate overall physical activity and physical activity intensity, we demonstrate that intensity may supersede overall physical activity in terms of reducing risk of CAD regardless of genetic risk. Our results within genetic risk strata largely agree with existing accelerometer-based studies, although we model overall physical activity and physical activity intensity linearly [10, 11].

### Strengths & limitations

This study is among the first to explore the association of genetic risk and accelerometer-measured overall physical activity and physical activity intensity with incident CAD. We use the strongest polygenic risk score and the largest sample of individuals with accelerometer measurements to date. By modeling physical activity continuously and objectively, we reduce the significant misclassification problems from discretizing subjective physical activity [38, 39]. The exploding commercial popularity of wrist-worn accelerometers has decreased the relevance of current physical activity standards for the population relying on these devices [40–42]. The current standards do not account for incidental physical activity, or physical activity performed as part of one’s normal activities, which means accelerometer-measured physical activity may make users appear falsely adherent to current guidelines. Studies relying on accelerometer-measured physical activity can close this gap [40].

The UK Biobank sample is disproportionately White and affluent relative to the general population and the sample who responded to take place in the accelerometer study represents further selection bias. However, previous studies have found in terms of physical activity, this cohort appears representative of the general population [43]. The self-reported covariates are measured at different times than accelerometer wear. Accelerometer wear occurred over seven days, which makes it cross-sectional, although we validate this against two waves of subjective physical activity in **S15 Fig in the S1 File,** which found a stronger correlation between more recent subjective physical activity and accelerometer wear. Previous studies have shown reactivity, or a behavioral response to accelerometer wear, may bias measured overall physical activity, although not MVPA [44]. While follow-up for CAD lasted for 6.8 years, this brief snapshot of physical activity could still mislead if a participant’s typical physical activity differs substantially from the week studied. More sophisticated machine learning methods can better discriminate between activity types and studies have shown our method of segregating percent MVPA is prone to misclassification [45, 46]. However, the potential for collinearity between overall physical activity and physical activity intensity persists given a greater level of MVPA translates into more overall PA. Wrist-worn accelerometers have limited ability to capture all physical activity, with housework, cycling, and weightlifting especially poorly captured [47, 48]. Because physical activity is not determined randomly, unmeasured confounding exists. We mitigate this concern by adjusting for related health behavioral factors, socioeconomic status, season of wear, and by performing sensitivity analyses adjusting for potential mediators.

### Conclusion

High genetic risk and low levels of physical activity volume and intensity were associated with large increases in incident CAD. This study showed physical activity is beneficial regardless of an individual’s underlying genetic risk and that genetic risk does not determine an individual’s fate regarding CAD [49]. This has important public health implications because it suggests that older adults have a significant ability to decrease their risk of CAD by engaging in more–and particularly more intense–physical activity. Older adults are half as likely to meet the guidelines for MVPA than adults aged 18 to 25 and objective physical activity tends to decline continuously across a person’s life [50, 51]. This study suggests that regardless of an individual’s underlying genetic risk or age, high levels of overall physical activity and MVPA–which can be reached by taking a brisk walk–remain protective against CAD.

## Supporting information

S1 File (DOCX)
