# Supplementary material for: Joint association of genetic risk and accelerometer-measured physical activity with incident coronary artery disease in the UK biobank cohort
Source: PLoS One. 2024 Jun 13;19(6):e0304653. doi: 10.1371/journal.pone.0304653 (PMC11175526; doi:10.1371/journal.pone.0304653)
Supplement: S1 File — (DOCX) [file pone.0304653.s001.docx]

**Supporting Information**

**Analyses using Physical Activity Energy Expenditure (PAEE) for volume and percent moderate-to-vigorous (% MVPA) physical activity for intensity**

Following Dempsey *et al*., we used a formula shown in **S1 Table** to convert these categorical midpoints of ENMO from dominant wrist-worn accelerometer data into instantaneous physical activity energy expenditure (PAEE).^1^ This measure was validated in free-living populations by both doubly labeled water and a combined heart rate monitor and trunk acceleration, the gold and silver standards of physical activity energy expenditure measurement, respectively.^2–4^ PAEE serves as the measure of physical activity volume in kJ/kg/day for our sensitivity analysis. In order to calculate physical activity intensity for the sensitivity analysis, we categorized physical activity above 125 milligravities as moderate-to-vigorous physical activity (MVPA) and then divided this value by total PAEE and multiplied by 100 to yield the percentage of PAEE from MVPA (percent MVPA).^1,2,5,6^

**S1 Table.** Equation Converting ENMO into PAEE and Derivation of Percent MVPA

| **Physical Activity Exposure** | **Definition** |
| --- | --- |
| Physical Activity Energy Expenditure (PAEE) | Following the work of Demspey, *et al*. (11), we apply the quadratic equation from White, *et al.* (21) to convert ENMO from wrist-worn accelerometer on dominant hand to PAEE with:  $PAEE= -10.58+1.1176*\left( 1.5+0.8517*x \right)+2.9148*\sqrt{\left( 1.5+0.8517*x \right)}-0.00059277*(1.5+0.8517*x)^{2}$  where x is the midpoint of one of UK Biobank’s predefined categories in milligravities. We then convert from J/min/kg into J/kg/day by multiplying by 1.44. We then sum over all of the intervals to get cumulative PAEE. |
| Percent MVPA | We divide PAEE spent above 125 mgs (equivalent to 3 METs) by overall PAEE to get the proportion of PAEE from moderate-to-vigorous physical activity and multiply this by 100 to yield the percent MVPA. |

**S1 Figure:** Overview of Physical Activity Volume (as PAEE) and Genetic Susceptibility Results


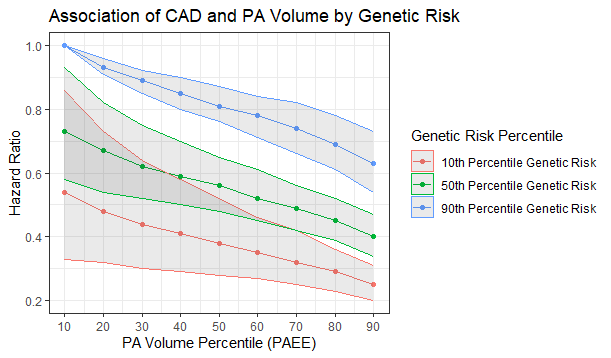


**S2 Figure.** Overview of Physical Activity Intensity (as % MVPA) and Genetic Susceptibility Results


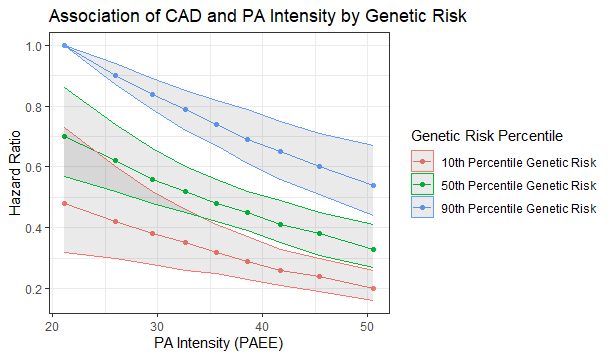


The central results for physical activity volume are virtually identical when using either ENMO or PAEE. Specifically, an individual at the 90^th^ percentile of physical activity volume faces a 37% lower hazard and a 39% lower hazard using PAEE and ENMO, respectively, at the same stratum of genetic risk. An individual at the 90^th^ percentile of PAEE and the 10^th^ percentile of genetic risk faces a 75% lower hazard of CAD than an individual at the 10^th^ percentile of PAEE and the 90^th^ percentile of genetic risk for both measures. Physical activity intensity measured as % MVPA has a slightly lower association with CAD than when it is measured as minutes per day of MVPA. An individual at the 90^th^ percentile of physical activity intensity faces a 46% lower hazard of CAD if measured as % MVPA compared to a 59% decrease if measured as minutes per day of MVPA. An individual at the 90^th^ percentile of physical activity intensity and 10^th^ percentile of genetic risk faces an 80% lower hazard of CAD compared to an individual at the 10^th^ percentile of physical activity intensity and 90^th^ percentile of genetic risk if using % MVPA compared to an 86% lower hazard using MVPA minutes per day.

**S3 Figure.** Correlations between different physical activity volume and intensity measures

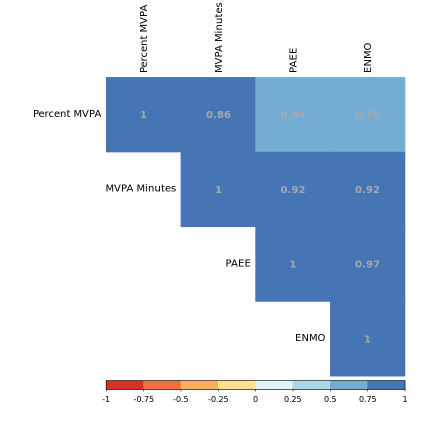


**S4 Figure.** Kaplan-Meier survival estimates for main sample


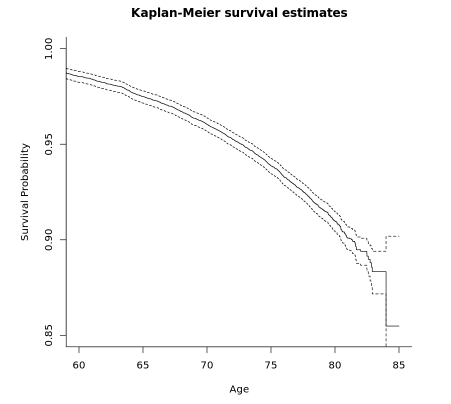


**S2 Table.** Definitions and Conversions for Covariates in Model 1

| **Covariate** | **Final Definition** | **UK Biobank Definition** | **Conversion** |
| --- | --- | --- | --- |
| Smoking Status | Categorical Variable with categories of Current, Never, and Previous following UK Biobank definition. | Categories of Current, Never, and Previous following UK Biobank definition. Derived from Current tobacco smoking and Past tobacco smoking fields. | None |
| Educational Attainment | Categorical variable with University Degree, Other Degree, and No Degree as levels. | Question asked: “which of the following qualifications do you have?” | Converted to University if “College or University Degree” selected, “None” if “None of the above” selected, and “Other” otherwise. |
| Employment Status | Binary variable with employment = 1 and other = 0. | Question asked: “which of the following describes your current situation?” | Converted to binary variable that equals 1 if “in paid employment or self-employed” and 0 if not. |
| Mobility Problems | Binary variable with mobility problems = 1 and no mobility problems = 0. | Question asked: “Please click the ONE box that best describes your health TODAY.” | Converted to binary variable that equals 1 if they indicate any issues walking and 0 otherwise. |
| Parental History of Heart Disease | Binary variable with existence of history = 1 and no history = 0. | Question asked: “Has/did your mother ever suffer from?” and “Has/did your father ever suffer from?” | Converted to binary variable that equals 1 if they indicate the mother OR father suffered from heart disease and 0 otherwise. |
| Weekly Processed Consumption | Numeric variable on frequency of processed meat consumption. | Question asked: “How often do you eat processed meats?” and lists options as never, less than once a week, once a week, 2-4 times a week, 5-6 times a week, once or more daily. | Converted to numeric variable with never = 0, less than once a week = 0.5, once a week = 1, 2-4 times a week = 3, 5-6 times a week = 5.5, once or more daily = 7. |
| Fruit & Vegetable Consumption Quartile | Quartiles from 1 to 4 denoting total fruit and vegetable consumption. | Composite of four total questions. Combined cooked and raw vegetable intake “On average how many heaped tablespoons of COOKED(RAW) vegetables would you eat per day?” Combined dried and fresh fruit intake “On average how many pieces of DRIED(FRESH) fruit would you eat per day?” | Added fruit total and vegetable totals together and then split these totals into quartiles. |
| Weekly Oily Fish Consumption | Numeric variable on frequency of oily fish consumption per week. | Question asked: “How often do you eat oily fish?” and lists options as never, less than once a week, once a week, 2-4 times a week, 5-6 times a week, once or more daily. | Converted to numeric variable with never = 0, less than once a week = 0.5, once a week = 1, 2-4 times a week = 3, 5-6 times a week = 5.5, once or more daily = 7. |
| Weekly Alcohol Consumption | Numeric variable on frequency of alcohol consumption per week. | Question asked: “about how often do you drink alcohol?” and lists daily or almost daily, three or four times a week, once or twice a week, one to three times a month, special occasions only, never. | Converted to numeric variable with never = 0, daily or almost daily = 7, three or four times a week= 3.5, once or twice a week = 1.5, one to three times a month = 0.4, special occasions only = 0.03. |
| Added Salt Intake | Factor variable with four levels never/rarely, sometimes, usually, always. | Question asked: “do you add salt to your food?” with options Never/rarely, sometimes, usually, always. | None |
| Season of Wear | Factor variable coded as Fall, Spring, Winter, or Summer based on date range. | Start time of wear denotes the date they began wearing an accelerometer. | Derived season ranges based on “Start time of wear” variable. |

**S3 Table.** Model 1 - controlling for full set of covariates in main analyses (Exposures Standardized)

| **Exposure** | **Hazard Ratio** |
| --- | --- |
| Standardized ENMO | HR = 0.81 (95% CI: 0.76 to 0.87) |
| Standardized PGS (in ENMO eqtn) | HR = 1.52 (95% CI: 1.43 to 1.61) |
| Standardized MVPA | HR = 0.70 (95% CI: 0.60 to 0.81) |
| Standardized PGS (in MVPA eqtn) | HR = 1.52 (95% CI: 1.43 to 1.61) |
| Standardized ENMO (in MVPA eqtn) | HR = 1.13 (95% CI: 0.98 to 1.30) |

**S4 Table.** Model 0 - controlling for age and biological sex (Exposures Standardized)

| **Exposure** | **Hazard Ratio** |
| --- | --- |
| Standardized ENMO | HR = 0.79 (95% CI: 0.74 to 0.84) |
| Standardized PGS (in ENMO eqtn) | HR = 1.55 (95% CI: 1.46 to 1.64) |
| Standardized MVPA | HR = 0.69 (95% CI: 0.59 to 0.80) |
| Standardized PGS (in MVPA eqtn) | HR = 1.55 (95% CI: 1.46 to 1.64) |
| Standardized ENMO (in MVPA eqtn) | HR = 1.09 (95% CI: 0.95 to 1.25) |

**S5 Table.** Overview of Physical Activity Volume (as ENMO) and Polygenic Risk by Decile

| ^ENMO^  _Stand PGS_ | 10^th^  (18.75 mgs) | 20^th^  (21.43 mgs) | 30^th^  (23.46 mgs) | 40^th^  (25.27 mgs) | 50^th^  (27.07 mgs) | 60^th^  (28.97 mgs) | 70^th^  (31.18 mgs) | 80^th^  (33.97 mgs) | 90^th^  (38.29 mgs) |
| --- | --- | --- | --- | --- | --- | --- | --- | --- | --- |
| 90^th^  (1.24 units) | REF | 0.93  (0.92-0.96) | 0.89  (0.86-0.92) | 0.85  (0.81-0.90) | 0.81  (0.76-0.87) | 0.77  (0.71-0.84) | 0.73  (0.66-0.81) | 0.68  (0.60-0.77) | 0.61  (0.52-0.72) |
| 80^th^  (0.81 units) | 0.91  (0.84-0.99) | 0.84  (0.78-0.91) | 0.80  (0.74-0.85) | 0.76  (0.71-0.81) | 0.72  (0.67-0.78) | 0.68  (0.63-0.74) | 0.64  (0.58-0.71) | 0.59  (0.53-0.67) | 0.53  (0.45-0.62) |
| 70^th^  (0.50 units) | 0.85  (0.73-0.98) | 0.78  (0.69-0.89) | 0.74  (0.66-0.82) | 0.70  (0.63-0.77) | 0.66  (0.60-0.73) | 0.62  (0.56-0.69) | 0.58  (0.52-0.65) | 0.54  (0.47-0.61) | 0.47  (0.40-0.55) |
| 60^th^  (0.24 units) | 0.80  (0.66-0.97) | 0.73  (0.62-0.87) | 0.69  (0.59-0.80) | 0.65  (0.56-0.75) | 0.61  (0.54-0.70) | 0.58  (0.51-0.66) | 0.54  (0.47-0.61) | 0.49  (0.43-0.56) | 0.43  (0.37-0.50) |
| 50^th^  (0.00 units) | 0.75  (0.59-0.96) | 0.69  (0.56-0.86) | 0.64  (0.53-0.78) | 0.61  (0.51-0.72) | 0.57  (0.48-0.67) | 0.54  (0.46-0.62) | 0.50  (0.43-0.58) | 0.45  (0.39-0.52) | 0.39  (0.33-0.46) |
| 40^th^  (-0.26 units) | 0.71  (0.53-0.96) | 0.65  (0.50-0.84) | 0.60  (0.48-0.76) | 0.57  (0.46-0.70) | 0.53  (0.44-0.65) | 0.50  (0.42-0.60) | 0.46  (0.39-0.54) | 0.42  (0.36-0.49) | 0.36  (0.30-0.43) |
| 30^th^  (-0.52 units) | 0.67  (0.48-0.95) | 0.61  (0.45-0.83) | 0.57  (0.43-0.75) | 0.53  (0.41-0.68) | 0.49  (0.39-0.62) | 0.46  (0.37-0.57) | 0.42  (0.35-0.51) | 0.38  (0.32-0.46) | 0.33  (0.27-0.39) |
| 20^th^  (-0.83 units) | 0.63  (0.42-0.94) | 0.56  (0.39-0.81) | 0.52  (0.38-0.72) | 0.49  (0.36-0.66) | 0.45  (0.34-0.59) | 0.42  (0.33-0.54) | 0.39  (0.31-0.48) | 0.35  (0.28-0.42) | 0.29  (0.24-0.35) |
| 10^th^  (-1.27 units) | 0.57  (0.35-0.93) | 0.51  (0.33-0.79) | 0.47  (0.31-0.69) | 0.43  (0.30-0.62) | 0.40  (0.29-0.56) | 0.37  (0.27-0.50) | 0.34  (0.26-0.44) | 0.30  (0.24-0.38) | 0.25  (0.20-0.31) |

**S6 Table.** Overview of Physical Activity Intensity (as minutes/day of MVPA) and Polygenic Risk by Decile

| ^MVPA Mins^  _Stand PGS_ | 10^th^  (31.68 mins/day) | 20^th^  (41.76 mins/day) | 30^th^  (51.84 mins/day) | 40^th^  (59.04 mins/day) | 50^th^  (67.68 mins/day) | 60^th^  (76.32 mins/day) | 70^th^  (87.84 mins/day) | 80^th^  (100.80 mins/day) | 90^th^  (120.96 mins/day) |
| --- | --- | --- | --- | --- | --- | --- | --- | --- | --- |
| 90^th^  (1.24 units) | REF | 0.91  (0.87-0.94) | 0.82  (0.75-0.89) | 0.76  (0.68-0.85) | 0.70  (0.60-0.81) | 0.64  (0.54-0.77) | 0.57  (0.46-0.72) | 0.51  (0.38-0.67) | 0.41  (0.29-0.60) |
| 80^th^  (0.81 units) | 0.88  (0.83-0.92) | 0.79  (0.74-0.83) | 0.71  (0.65-0.77) | 0.66  (0.59-0.74) | 0.60  (0.52-0.70) | 0.55  (0.46-0.66) | 0.49  (0.39-0.61) | 0.42  (0.32-0.57) | 0.34  (0.24-0.50) |
| 70^th^  (0.50 units) | 0.79  (0.73-0.87) | 0.71  (0.66-0.77) | 0.64  (0.58-0.70) | 0.59  (0.52-0.66) | 0.54  (0.46-0.62) | 0.49  (0.40-0.59) | 0.43  (0.34-0.54) | 0.37  (0.28-0.50) | 0.30  (0.21-0.44) |
| 60^th^  (0.24 units) | 0.73  (0.65-0.82) | 0.65  (0.59-0.73) | 0.65  (0.59-0.73) | 0.54  (0.47-0.61) | 0.49  (0.42-0.57) | 0.44  (0.37-0.53) | 0.39  (0.31-0.49) | 0.33  (0.25-0.45) | 0.27  (0.18-0.39) |
| 50^th^  (0.00 units) | 0.68  (0.58-0.78) | 0.60  (0.53-0.69) | 0.53  (0.47-0.61) | 0.49  (0.43-0.57) | 0.44  (0.38-0.52) | 0.40  (0.33-0.49) | 0.35  (0.28-0.45) | 0.30  (0.22-0.41) | 0.24  (0.16-0.35) |
| 40^th^  (-0.26 units) | 0.63  (0.52-0.75) | 0.56  (0.48-0.65) | 0.49  (0.42-0.57) | 0.45  (0.39-0.53) | 0.41  (0.34-0.48) | 0.37  (0.30-0.45) | 0.32  (0.25-0.41) | 0.27  (0.20-0.37) | 0.21  (0.14-0.32) |
| 30^th^  (-0.52 units) | 0.58  (0.47-0.71) | 0.51  (0.42-0.61) | 0.45  (0.38-0.53) | 0.41  (0.35-0.49) | 0.37  (0.31-0.44) | 0.33  (0.27-0.41) | 0.29  (0.22-0.37) | 0.24  (0.18-0.33) | 0.19  (0.13-0.29) |
| 20^th^  (-0.83 units) | 0.52  (0.41-0.67) | 0.46  (0.37-0.57) | 0.40  (0.33-0.49) | 0.37  (0.31-0.45) | 0.33  (0.27-0.40) | 0.30  (0.24-0.37) | 0.25  (0.20-0.33) | 0.22  (0.16-0.30) | 0.17  (0.11-0.25) |
| 10^th^  (-1.27 units) | 0.46  (0.34-0.62) | 0.40  (0.31-0.52) | 0.35  (0.28-0.44) | 0.32  (0.25-0.39) | 0.28  (0.23-0.35) | 0.25  (0.20-0.32) | 0.21  (0.16-0.28) | 0.18  (0.13-0.25) | 0.14  (0.09-0.21) |

**S5 Figure.** Overview of Physical Activity Volume and Genetic Susceptibility Results (First Year Excluded)


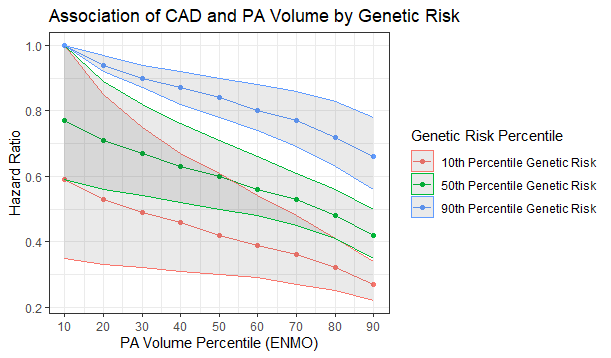


**S6 Figure.** Overview of Physical Activity Intensity and Genetic Susceptibility Results (First Year Excluded)


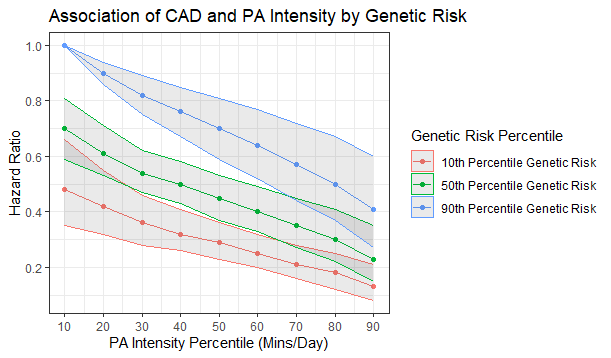


**S7 Figure.** Overview of Physical Activity Volume and Genetic Susceptibility Results (MICE imputation)


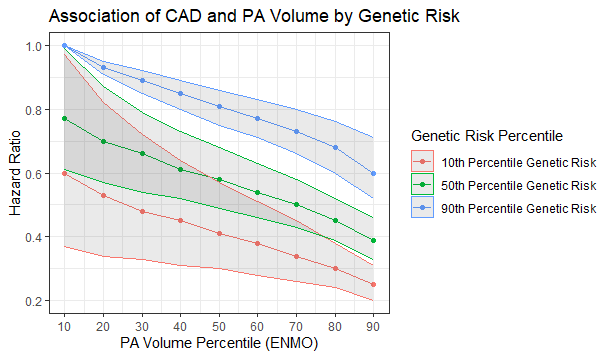


**S8 Figure.** Overview of Physical Activity Intensity and Genetic Susceptibility Results (MICE imputation)


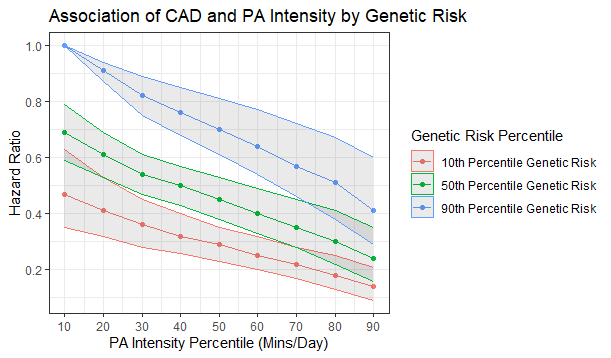


**S9 Figure.** Add BMI, Sleep Duration, Medication use to Model 1 for Physical Activity Volume and Genetic Susceptibility


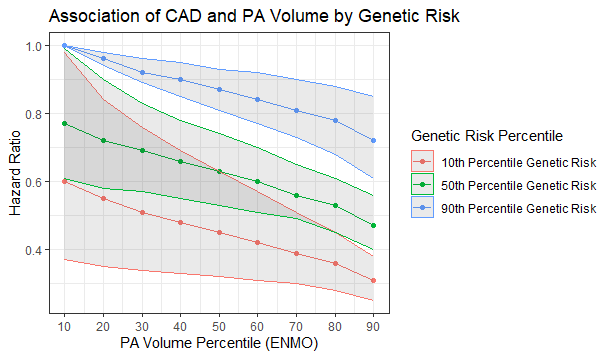


**S10 Figure.** Add BMI, Sleep Duration, Medication use to Model 1 for Physical Activity Intensity and Genetic Susceptibility


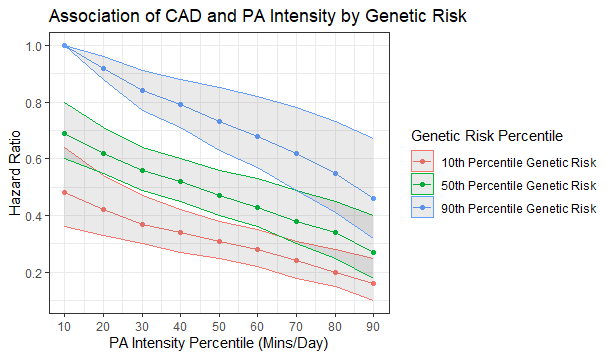


**S11 Figure.** Add BMI, Sleep Duration, Medication use, whether individual is physically active in occupation to Model 1 for Physical Activity Volume and Genetic Susceptibility


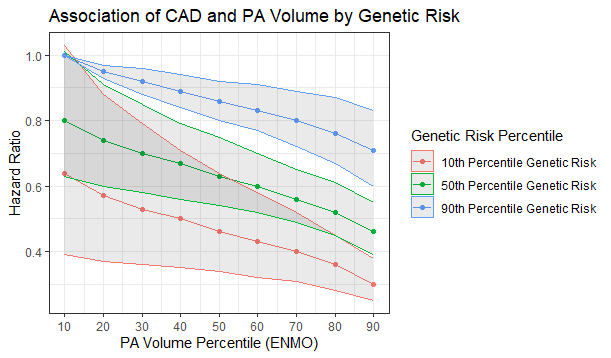


**S12 Figure.** Add BMI, Sleep Duration, Medication use, whether individual is physically active in occupation to Model 1 for Physical Activity Intensity and Genetic Susceptibility


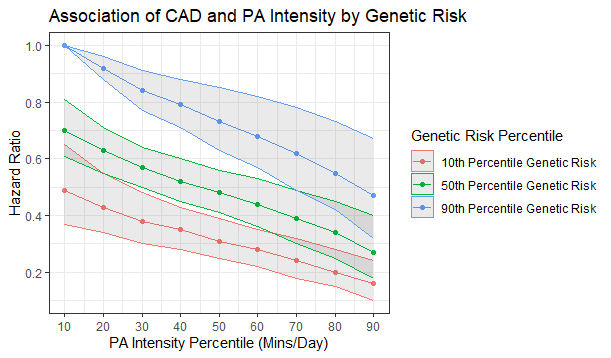


**S13 Figure.** Overview of Physical Activity Volume and Genetic Susceptibility Results (Sex Stratified)

| 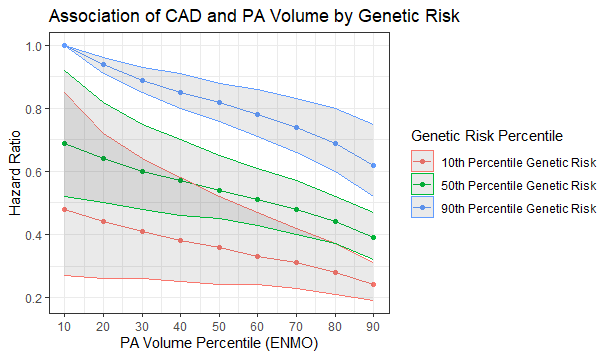  MALE |
| --- |
| 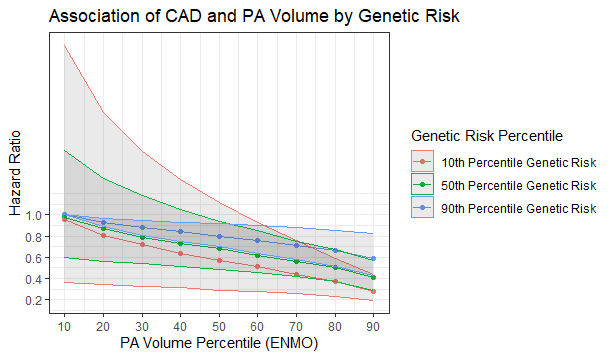  FEMALE |

**S14 Figure.** Overview of Physical Activity Intensity and Genetic Susceptibility Results (Sex Stratified)

| 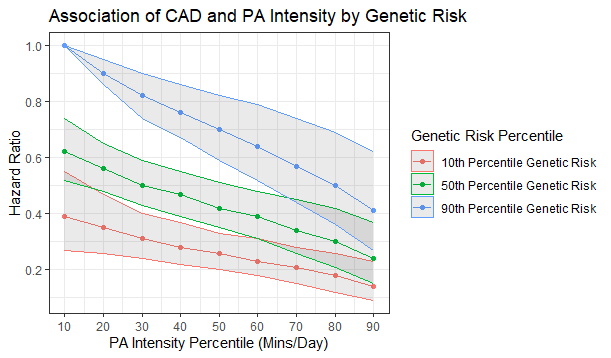  MALE |
| --- |
| 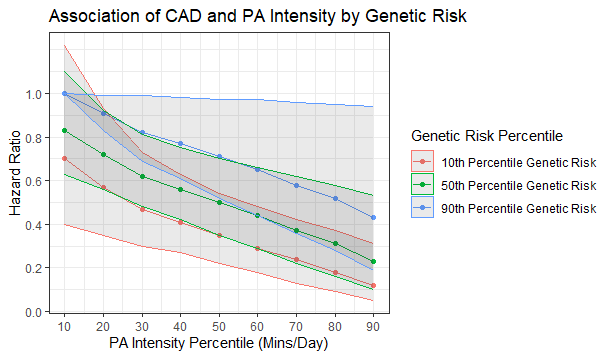  FEMALE |

**S15 Figure.** Objective PA vs Longitudinal Subjective PA Correlation

| 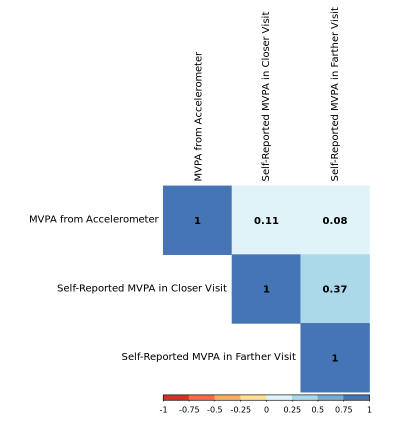 |
| --- |
| *While correlation between self-reported MVPA and MVPA from accelerometer are low, this correlation changes relatively little between closer or farther visit from accelerometer wear start date and is in line with low correlations even between self-reported MVPA and MVPA from accelerometers measured contemporaneously. |

**Supporting Information References**

1. Dempsey PC, Rowlands A V, Strain T, et al. Physical activity volume, intensity, and incident cardiovascular disease. *Eur Heart J*. 2022;43(46):4789-4800. doi:10.1093/EURHEARTJ/EHAC613

2. White T, Westgate K, Hollidge S, et al. Estimating energy expenditure from wrist and thigh accelerometry in free-living adults: a doubly labelled water study. *Int J Obes 2019 4311*. 2019;43(11):2333-2342. doi:10.1038/s41366-019-0352-x

3. White T, Westgate K, Wareham NJ, Brage S. Estimation of Physical Activity Energy Expenditure during Free-Living from Wrist Accelerometry in UK Adults. *PLoS One*. 2016;11(12):e0167472. doi:10.1371/JOURNAL.PONE.0167472

4. Villars C, Bergouignan A, Dugas J, et al. Validity of combining heart rate and uniaxial acceleration to measure free-living physical activity energy expenditure in young men. *J Appl Physiol*. 2012;113(11):1763-1771. doi:10.1152/JAPPLPHYSIOL.01413.2011/ASSET/IMAGES/LARGE/ZDG0221203780002.JPEG

5. Ramakrishnan R, Doherty A, Smith-Byrne K, et al. Accelerometer measured physical activity and the incidence of cardiovascular disease: Evidence from the UK Biobank cohort study. *PLOS Med*. 2021;18(1):e1003487. doi:10.1371/JOURNAL.PMED.1003487

6. Strain T, Wijndaele K, Dempsey PC, et al. Wearable device measured physical activity and future healthrisk. *Nat Med*. 2020;26(9):1385. doi:10.1038/S41591-020-1012-3
